# Beethoven recordings reviewed: a systematic method for mapping the content of music performance criticism

**DOI:** 10.3389/fpsyg.2015.00057

**Published:** 2015-02-17

**Authors:** Elena Alessandri, Victoria J. Williamson, Hubert Eiholzer, Aaron Williamon

**Affiliations:** ^1^School of Music, Lucerne University of Applied Sciences and ArtsLucerne, Switzerland; ^2^Centre for Performance Science, Royal College of MusicLondon, UK; ^3^Department of Music, University of SheffieldSheffield, UK; ^4^School of Advanced Study, University of LondonLondon, UK; ^5^Department of Research and Development, Conservatory of Southern SwitzerlandLugano, Switzerland

**Keywords:** music criticism, performance, aesthetic judgment, Beethoven, recordings

## Abstract

Critical reviews offer rich data that can be used to investigate how musical experiences are conceptualized by expert listeners. However, these data also present significant challenges in terms of organization, analysis, and interpretation. This study presents a new systematic method for examining written responses to music, tested on a substantial corpus of music criticism. One hundred critical reviews of Beethoven’s piano sonata recordings, published in the *Gramophone* between August 1934 and July 2010, were selected using in-depth data reduction (qualitative/quantitative approach). The texts were then examined using thematic analysis in order to generate a visual descriptive model of expert critical review. This model reveals how the concept of evaluation permeates critical review. It also distinguishes between two types of descriptors. The first characterizes the performance in terms of specific actions or features of the musical sound (musical parameters, technique, and energy); the second appeals to higher-order properties (artistic style, character and emotion, musical structure, communicativeness) or assumed performer qualities (understanding, intentionality, spontaneity, sensibility, control, and care). The new model provides a methodological guide and conceptual basis for future studies of critical review in any genre.

## INTRODUCTION

The tradition of music written response has a long history of communicating listener reactions and providing performance descriptions and assessments. Examples include examination reports and competition rankings, booklets and concert notes, and reviews by peers and professional critics. These responses have an impact on musicians’ lives and careers and offer a direct source of feedback for performers throughout their musical development.

One of the best known forms of music written response is the grading of performance, done either holistically or through a segmented, pre-defined scheme. This written response, commonly used in educational and competitive contexts, has been the object of a substantial corpus of research. Studies have improved understanding of associated feedback, of its reliability and consistency (Fiske, 1977; Wapnick et al., 1993; Bergee, 1997, 2003; Thompson and Williamon, 2003; Kinney, 2009; Wrigley and Emmerson, 2013), and of the different performance elements, including the non-musical, which affect evaluative judgments (McPherson and Schubert, 2004; Ryan et al., 2006; Vines et al., 2006; Schutz and Lipscomb, 2007; Thompson, 2007; Thompson et al., 2007; Griffiths, 2010; Lehmann and Kopiez, 2013; Platz and Kopiez, 2013).

There is, however, another form of music written response that is also wide-spread and important to the performing musician, yet about which we know little by comparison: critical review. This medium can be described as a form of professional writing that encompasses activities like evaluation, description, elucidation, classification, contextualization, interpretation, and/or analysis of live or recorded performances (Carroll, 2009, pp. 13–14).

Critical review that focuses on performance, rather than on the work performed, has been the fashion since the turn of the twentieth century (Monelle, 2002; Alessandri et al., 2014). Despite the availability of representative material and its impact on musicians’ careers, there has been little structured enquiry into the way expert music critics make sense of their experience of performances, how they structure and verbalize their musical impressions.

The only relevant study to our knowledge was carried out by Van Venrooij and Schmutz (2010), who analyzed 122 critical reviews of popular music albums. Their aim was to observe differences in the use of high-art and popular aesthetic criteria in different countries (USA, Germany, and Netherlands). Their findings showed distinctive patterns for the three countries, with high-art criteria significantly more present in German compared with Dutch or American reviews. Here, however, researchers used a deductive approach, applying a pre-defined categorization of what constitutes high-art or popular aesthetic criteria. No study to date has used inductive methods to produce a valid, comprehensive model of critical review content, in any musical genre.

The question of music review content can be framed against a wider issue; a lack of agreement concerning the basic components of art criticism. One fundamental issue concerns the importance of an evaluative component. The understanding of art criticism as a form of evaluation is grounded in “long standing historical trends in the practice of criticism” (Carroll, 2009, p. 16), and this assumption underpins seminal works in the field (Calvocoressi, 1923; Newman, 1925; Walker, 1968; Cone, 1981). However, in recent years the notion of critical review as ‘evaluation’ has found opposition. Elkins (2003) stated that the second half of the twentieth century witnessed a ‘denaturalization’ of art criticism into a non-judgmental, descriptive and evocative exercise. Danto (in Rubinstein, 2006) argues that evaluating art is an activity that has been removed from the responsibilities of the critic and which rather lies in the hands of curators and managers.

This debate regarding the role of evaluation in critical review seems to resonate with a survey of 181 music critics (Conrad et al., 2005). In this study, less than half of the critics (45%) stated that evaluation was of great importance when reviewing. The rendering of a personal judgment was given priority compared with theorizing and providing historical or other background. However, it was ranked lower in importance than describing purpose, location, and feeling, describing the aural experience, and creating a piece of writing with literary value (p. 18).

This wider debate on the role of such a basic critical review component as evaluation underlines the importance of understanding how expert music critics convey their musical impressions to readers. Despite critical review being one of the most common professional and commercial forms of music written response, no studies to date have broached the key question of how music performance is reviewed by experts. A better understanding of critical review could be of great value for musicians as they learn and prepare for performance, thereby contributing important insights to music education. The present study offers a first step toward this aim. It presents a systematic method for analyzing the rich and detailed content of critical review data, tested on a large sample of recorded performance criticism from the classical music repertoire. Our research question was simple: what do experts write about when reviewing a recorded musical performance?

## MATERIALS AND METHODS

### SOURCE MATERIAL

For the present study, it was necessary to produce a representative corpus of best practice in classical music critical review apt for systematic and detailed investigation. Such a corpus needed to be large and varied enough to allow a valid overview of critical practice and yet focused enough to permit an in-depth investigation of its content.

Following the assumption that cultural background and repertoire may reasonably affect the review content, we restricted the investigation to one source material and to one delimited repertoire. This decision, although limiting the generalizability of results, allowed for a more in-depth examination of the material. The initial sample from which the corpus was eventually extracted thus entailed all reviews of recordings of Beethoven’s piano sonatas published in the British magazine *Gramophone* between 1923 (when the magazine was founded) and 2010 (the beginning of the study). This sample comprised 845 texts, on average 411.74 words long (Alessandri et al., 2014).

The *Gramophone* is one of the oldest magazines to contain reviews of classical music recordings. Its authority as a leader in reviews of recorded performance, combined with the unique coverage it offers (over 90 years) and the availability of this material in digital format (the whole archive was digitalized and made public in 2009), made for a unique opportunity for an in-depth investigation of best practice in performance criticism.

Within the material available in the *Gramophone*, the choice of Beethoven’s piano sonata recordings as the sample of repertoire was made due to these sonatas’ place among the most often performed pieces of classical music in pianists’ standard repertoire. Thus, reviews of Beethoven sonata recordings allowed access to a vast and rich amount of critical material and permitted insight into critical practice that has a direct relevance for a majority of pianists and piano pedagogues.

### ANALYTICAL APPROACH

The study of unstructured text as typified by our sample is open to many analytical approaches. A person-centered, interpretative approach offers the best way to capture the rich context-related meanings within the music critical review texts. The principal alternative, a positivist computer-assisted approach is not appropriate in the present case. In fact, critical review texts are not simple enough to allow the application of limited templates. Moreover, the wording can be ambiguous (‘slow *movement* of the sonata’ versus ‘the forward *movement* and dramatic tension produced by the pianist’) and metaphorical in nature. As such, the text data are not specified explicitly and are not locally focused: characteristics necessary to the successful application of computer-assisted techniques (Feldman and Sanger, 2007).

Two challenges of the person-centered approach are its dependency on interpretation and its time-consuming nature. In the present study we employed the Applied Thematic Analysis approach (ATA; Guest et al., 2012) in answer to these challenges. ATA is a practice-based, mixed methods approach to qualitative analysis. Qualitative text analysis is at the core of ATA, but this is accompanied by the principle that data must be paramount in deciding at any stage what analytical method to use, without excluding *a priori* any theoretical and epistemological approach. ATA is linked to grounded theory in its emphasis on supporting claims by means of evidence grounded in data and in its need for an interpretation of data that is always coherent with the actual texts at hand. The approach is thus systematic (e.g., in the codebook development, code application and data reduction) and iterative.

The main feature that distances ATA from grounded theory is the fact that ATA allows the use of quantification and data reduction techniques, as long as they are complementary to the qualitative analysis and appropriate to the analytical purpose. Given its hybrid nature, ATA offers a solution that maximizes the level of in-depth, qualitative analysis while allowing for the examination of large sets of textual data. Following the ATA approach, in the present study a new analysis protocol was applied that combined data reduction procedures with qualitative thematic analysis techniques. Firstly, the *Gramophone* sample was reduced from the initial sample to a representative corpus by a process of data reduction. Then, the selected corpus was qualitatively analyzed by applying a protocol previously adopted by Williamson et al. (2012) and Williamson and Jilka (2013) in their studies of musical experiences.

### DATA REDUCTION

Following the ATA approach (Namey et al., 2008; Guest et al., 2012) preliminary data reduction allowed for the selection of a manageable, representative corpus of material for subsequent qualitative thematic analysis. This entailed the following steps:

(i) A thick-grained qualitative text analysis of a selection of reviews (*n* = 63) to produce a categorization of the topics discussed (Alessandri et al., 2011);
(ii) An estimation of this categorization for the whole dataset, using the R package ReadMe^1^: a learning machine, text mining application that estimates the distribution of pre-defined categories among texts (Hopkins and King, 2010);
(iii) A qualitative analysis of critics’ vocabulary, with vocabulary organized into different semantic categories;
(iv) A comparison of the use of these categories between critics and in different time periods, using the Linguistic Inquiry and Word Count application (Tausczik and Pennebaker, 2010).

The first stages of the data reduction (i, ii) revealed that three main topics were discussed in the whole critical review sample (*N* = 845): *interpretation/performance* (53.50% of the text), *composition* (9.09%), and *recording* (16.73%). *Interpretation/performance* was clearly the predominant topic. Its presence increased over time, ranging from 36.38% during 1923–1950 to 60.17% during 1991–2010^2^. Given the large sections of text devoted to the discussion of interpretative issues, it was concluded that a relatively small number of reviews would provide a valid overview of this aspect of critical review. Furthermore, the aim of the present study was to explore the content of critical review with regard to the performance as opposed to the original composition or the recording process, therefore the subsequent thematic analysis focused on the review text that dealt with *interpretation/performance*.

In the next stage of data reduction (iii) a vocabulary of the critical review sample was compiled using the word cruncher function of the software Atlas.ti (version 6.1). This resulted in a list of 17,340 word types. This list was reduced by (a) narrowing the analysis to words that occurred more than five times in the whole dataset; and (b) sorting out function words and proper names. The remaining 2,503 word types were analyzed by the first author and grouped according to different semantic fields.

The grouping was carried out from bottom–up, letting the categories emerge from the list. However, the Reasoning Model, as it is described by Beardsley (1968, 1982), was taken as background to the analysis. According to this model, judgments of value can be supported by reasons, which explain why the judgment is true by means of appealing to qualities that are inherent in the artwork (Beardsley, 1968, p. 57). The Reasoning Model can thus be expressed in its simplest form through the formula: Performance P is good/bad or better/worse than Performance W, because it possesses feature F, where F is a feature that resides in the artwork. Based on this model, critics’ vocabulary was then divided in three main groups: (1) purely evaluative terms (words that comprise only an evaluative component but offer no description of the object being evaluated, see Bonzon, 2009); (2) words that indicate a reasoning process (e.g., despite, consequently, and yet, thus, therefore); and (3) all words that may suggest some kind of reason for the judgment^3^.

This resulted in 13 semantic categories of words, which were then uploaded on the LIWC software and computed for frequency in the sample (iv). This analysis revealed that the use of vocabulary was shaped more strongly by reviewer identity than by the review time period. *Gramophone* reviewers were compared (*n* = 10) who had an average activity period of over 20 years, the longest lasting 41 years (Alessandri et al., 2014). Over these periods, word patterns and weight given to semantic categories remained constant within critic, while differing significantly between critics (Kruskal–Wallis: *H*_6_ = 25.59, *p* = 0.086 between decades; *H*_10_ = 52.30, *p* = 0.037 between critics). Following this finding, it was decided that the selection of reviews for the corpus should be led by critic, rather than by review period, to better capture the richness of the data.

In the light of the data reduction analyses, 10 critical reviews were selected from each of the 10 most prolific critics in the original sample (**Table 1**) so as to maximize variability in terms of period, pianist, and sonatas reviewed. The 100 selected reviews in the final corpus (35,753 words, excluding titles, critics’ names and recording details) spanned August 1934 to July 2010, entailed 56 pianists, and comprised at least six reviews for each of Beethoven’s 32 sonatas^4^.

**Table 1:** Corpus selected for the in-depth thematic analysis.

Reviewer	Reviews (*Gramophone* issue, page)
Alec Robertson	Aug ’34, p. 29; Oct ’35, p. 18; Apr ’36, p. 18; Nov ’36, p. 17; Feb ’37, p. 19; Oct ’45, p. 16; Feb ’47, p. 8; Feb ’48, p. 23; Aug ’50, p. 23; Oct ’53, p. 22
Roger Fiske	Jul ’55, p. 44; Nov ’57, p. 17; Oct ’58, p. 65; Apr ’59, p. 64; Nov ’59, p. 67; Nov ’59, p. 68; Feb ’61, p. 48; Aug ’63, p. 31; Jul ’84, p. 41; Feb ’86, p. 52
Joan Olive Chissell	Mar ’69, p. 66; Jun ’69, p. 53; Feb ’70, p. 54; Dec ’70, p. 86; Jun ’71, p. 54; Mar ’72, p. 74; Mar ’75, p. 81; Oct ’80, p. 71; Feb ’83, p. 52; Jun ’92, p. 66
Andrew Porter	Jun ’54, p. 42; Feb ’59, p. 60; Oct ’54, p. 50; Oct ’54, p. 51; Feb ’55, p. 56; May ’56, p. 49; Nov ’56, p. 55; Jun ’57, p. 19; Sept ’57, p. 17; May ’58, p. 16
Stephen Plaistow	Dec ’61, p. 57; Jun ’62, p. 64; Jun ’63, p. 36; Mar ’64, p. 63; Mar ’65, p. 57; Jul ’66, p. 47; Aug ’79, p. 69; Mar ’88, p. 50; Oct ’89, p. 98; Jan ’02, p. 81
Richard Osborne	Apr ’82, p. 66; May ’83, p. 49; Dec ’83, p. 84; Aug ’86, p. 49; Mar ’93, p. 73; Sept ’95, p. 83; Nov ’95, p. 146; Feb ’96, p. 75; Nov ’00, p. 86; Nov ’04, p. 79
Malcolm MacDonald	Aug ’54, p. 39; Nov ’64, p. 52; Jan ’65, p. 59; Mar ’65, p. 57; Mar ’65, p. 58; Jan ’68, p. 84; Jan ’70, p. 56; May ’81, p. 92; Nov ’81, p. 82; Dec ’81, p. 84
David J. Fanning	Sept ’86, p. 84; Nov ’86, p. 78; Sept ’88, p. 80; Jun ’89, p. 64; Mar ’90, p. 69; Sept ’90, p. 116; Oct ’90, p. 116; Mar ’91, p. 85; Apr ’92, p. 111; Nov ’92, p. 152
Bryce Morrison	May ’93, p. 74; Feb ’02, p. 63; Dec ’02, p. 72; Mar ‘3, p. 63; Jan ’05, p. 76; May ’05, p. 104; Jun ’06, p. 71; Jun ’08, p. 81; Jul ’10, p. 77(i); Jul ’10, p. 77(ii)
Jed Distler	Oct ’05, p. 81; Oct ’09, p. 88; Dec ’05, p. 97; May ’06, p. 90; Sept ’06, p. 80; Nov ’06, p. 97; Apr ’07, p. 92; Jun ’07, p. 84; Sept ’07, p. 76; Dec ’08, p. 103

*The table reports names of the 10 selected critics and details of the single reviews (issue, page).*

### IN-DEPTH ANALYSIS

The in-depth, inductive analysis of the corpus that emerged from the data reduction phase was based on the protocol developed in Williamson et al. (2012) and Williamson and Jilka (2013).

The analysis focused on aspects of the performance discussed in reviews. Therefore, selected reviews were further pre-prepared by visually separating (highlighting) parts of the text concerning the performance from the rest [following the codebook developed in the data reduction analysis (i)]. Review text that did not concern the performance was not included in the analysis. To add validity to the analysis, the protocol involved the participation of two researchers (first and second authors) in the development of the codebook and an iterative process of text comparison and code revision. The two researchers were chosen so as to reflect the standpoints of two common categories of review readers: the professionally trained listener, who is familiar with the work discussed and comes to the repertoire with both knowledge and strong personal preferences (first author), and the more generally musically trained listener, who has a solid grasp of the musical vocabulary but is not necessarily familiar with the repertoire and the technicalities of the instrument reviewed (second author). This variety of perspectives was sought to permit the development of a model whose application and understanding could be open to a wider audience and would not require professional musical training.

The analysis involved three stages. In the initial stage, a subset of 10 reviews (one for each critic) was hand-coded by the two researchers independently, using line-by-line open coding. Each researcher then organized their codes into themes that summarized the content of the reviews. Emergent themes were then compared between the two researchers. To minimize subjectivity of interpretation, each researcher in turn explained a theme, justifying it by means of examples from the data and proposing a definition. Based on this, an agreed codebook of emergent themes was developed.

In the next stage, the main researcher applied the codebook to the whole dataset, revising themes and definitions only where appropriate. Atlas.ti 6.1 was used and segmentation of text was performed at clause level, with clause understood as the minimal independently meaningful text unit, consisting at least of a subject and a predicate. Codes were understood as non-mutually exclusive, thus each clause could be attached to several lower-level themes. The application of a clear principle for the segmentation of text during coding was important to ensure a meaningful comparison of themes in terms of frequency with which they were coded.

Hence, the sentence: “The section of the slow movement has a certain beauty which I feel Schnabel spoils by too dynamic a treatment” was coded as one segment, as: ***Evaluation***, *Beauty*, ***Authenticity***, ***Style***(see the first section of Results for use of bold, bold italic, and italic formatting). The sentence “The dynamic range is wonderful and a more than usually striking significance is given to the four-note phrase so reminiscent of the opening of the Fifth Symphony” was divided in two segments: “The dynamic range is wonderful” (codes: ***Musical Parameters***, *Dynamics*, **Evaluation**) and “a more than usually striking significance is given to the four-note phrase” (codes: ***Structure***, *Emphasis*). The last part of the sentence was not coded, since it describes the work performed without comments on its interpretation.

One new theme (**Evaluation**_*Taste*) emerged at this second stage of the coding process; this theme was discussed with the second coder and added to the theme codebook. After about one third of the documents were coded, saturation point was achieved. After this, no new themes were found and the whole dataset was analyzed using the completed codebook.

Finally, upon completion of the full coding stage, lists of quotes were retrieved for each theme separately (using the code output function of Atlas.ti) and analyzed. Reading quotes grouped per theme and outside their textual context permitted a more focused analysis, in which quotes within and between themes were compared in an iterative process to check for coding mistakes and clarify distinctions and relationships so as to maximize differentiation between and homogeneity within themes. Revisions to the codebook definitions and structure resulting from this analysis were discussed and agreed upon between the two researchers. In this stage a group of sub-themes emerged that seemed to share a focus on the agent of the performance. The heading Performer Qualities was created to refer to this *post hoc* summary of related sub-themes. At the end of this process, coding for the whole dataset was revised to adjust it to the newly emerged model. This led to the development of a visual descriptive model of critical review.

## RESULTS

Critical review emerged as a dense form of writing. The 100 reviews resulted in a total of 6,012 codes with an average density of 6.66 codes per clause. Density across critics ranged from 5.01 (MacDonald) to 9.76 codes per clause (Distler). The fact that codes were so closely spaced limited the kinds of analysis that could be run: thematic analysis usually enables the exploration of patterns of themes through the observation of co-occurrences between codes. The high number of codes per clause found in reviews made co-occurrence tables unusable.

Furthermore, the critics’ concise writing style, in which musically relevant qualities are often simply listed, assuming a shared understanding of their meaning and connotations, rendered it difficult to move the analysis to a deep interpretative level without losing connection with the data and entering the domain of speculation. What the present analysis permitted, however, was the creation of a comprehensive map of the topics discussed in critical review.

On completion of the analysis there were three superordinate theme families – Primary Descriptors, Supervenient Descriptors, and Evaluative Judgments, with 12 dominant themes, which comprised a further 33 sub-themes. Evaluative Judgments comprises comments on the value, importance, usefulness, or merit of the performance. This was the largest superordinate theme family, with 1,502 occurrences. This family also entailed the single largest and most widely spread dominant theme in the whole analysis, ***Evaluation*** (1,100 occurrences, found in 100% of reviews, see **Table 2**). Primary and Supervenient Descriptors entailed characterizations of the performance. Supervenient Descriptors was the more prominent family of the two and the most varied in the whole analysis, encompassing 1,404 occurrences and 20 sub-themes. Within this family, a group of sub-themes has been highlighted in the model (*Performer Emotion*, *Performer Character*, *Performer Style*, and *Performer Understanding*), that characterizes the performance focusing on its agent rather than on the performance itself, thus assigning qualities to the performer (Performer Qualities).

**Table 2:** Distribution of dominant (bold) and first level sub-themes across the 100 reviews and for each critic separately (10 reviews per critic).

		*Theme*	90*All reviews (N = 100)*	90*Robertson (n = 10)*	90*Fiske (n = 10)*	90*Chissell (n = 10)*	90*Porter (n = 10)*	90*Plaistow (n = 10)*	90*Osborne (n = 10)*	90*MacDonald (n = 10)*	90*Fanning (n = 10)*	90*Morrison (n = 10)*	90*Distler (n = 10)*
**Evaluative judgments**
		***Evaluation***	**100**	**10**	**10**	**10**	**10**	**10**	**10**	**10**	**10**	**10**	**10**
		*Affective*	89	7	10	9	9	9	10	7	10	9	9
		*Comparison*	63	4	8	5	8	4	7	1	7	9	10
		*Taste*	47	7	7	6	4	5	5	3	3	3	4
		*Clarity*	31	5	1	6	3	3	1	*0*	3	3	6
		*Success*	23	2	2	1	3	2	2	4	3	4	*0*
		*Beauty*	20	4	4	3	4	*0*	2	1	*0*	*0*	2
		***Authenticity***	**89**	**9**	**9**	**10**	**9**	**9**	**7**	**8**	**10**	**9**	**9**
		*Notation*	48	4	7	4	4	6	2	2	7	4	8
		*Historical*	11	*0*	2	3	1	1	1	2	*0*	1	*0*
		***Novelty***	**59**	**7**	**5**	**8**	**4**	**4**	**5**	**3**	**8**	**8**	**7**

**Supervenient descriptors**
		***Style***	**95**	**10**	**9**	**10**	**9**	**9**	**10**	**8**	**10**	**10**	**10**
		*Performer*	80	6	6	10	8	9	7	6	10	9	9
		*Historical*	23	1	4	4	2	3	2	*0*	3	3	1
		*Expressive*	17	*0*	3	5	2	2	*0*	*0*	*0*	1	4
		***Structure***	**85**	**8**	**7**	**9**	**10**	**8**	**10**	**5**	**9**	**9**	**10**
		*Journey*	48	3	5	4	4	7	7	3	3	6	6
		*Balance*	43	1	2	5	4	6	5	2	7	4	7
		*Emphasis*	24	5	2	1	1	4	3	*0*	2	2	4
		***Understanding***	**79**	**7**	**9**	**8**	**8**	**8**	**7**	**5**	**9**	**9**	**9**
		*Performer*	71	7	9	8	5	8	5	5	8	7	9
		***Character***	**79**	**6**	**8**	**8**	**8**	**6**	**9**	**5**	**10**	**9**	**10**
		*Performer*	23	1	1	2	1	1	4	*0*	7	5	1
		***Emotion***	**59**	**6**	**7**	**6**	**6**	**7**	**5**	**4**	**5**	**6**	**7**
		*Performer*	26	2	6	4	1	2	2	*0*	3	2	4
		***Dialog***	**52**	**2**	**5**	**4**	**6**	**7**	**5**	**3**	**6**	**7**	**7**

**Primary descriptors**
		***Musical Parameters***	**89**	**10**	**10**	**10**	**9**	**9**	**7**	**8**	**9**	**7**	**10**
		*Time*	73	7	9	10	6	8	5	5	8	5	10
		*Color*	41	4	2	5	4	5	5	*0*	6	1	9
		*Dynamics*	37	7	3	5	5	5	*0*	1	4	3	4
		*Articulation*	29	5	3	4	*0*	2	*0*	1	2	2	10
		*Rhythm*	26	3	*0*	4	4	4	3	2	3	2	1
		***Energy***	**72**	**4**	**7**	**9**	**7**	**8**	**7**	**5**	**6**	**9**	**10**
		*Tension*	11	*0*	*0*	2	*0*	2	3	*0*	1	*0*	3
		***Technique***	**61**	**6**	**6**	**9**	**4**	**4**	**7**	**4**	**7**	**5**	**9**
		*Virtuosity*	15	1	1	1	*0*	1	2	1	2	3	3

*Themes are treated as dichotomous variables: for each review, a theme was given the value of 1 if it occurred at least once in the text, a value of 0 if it did not occur in the text.*

**FIGURE 1:**
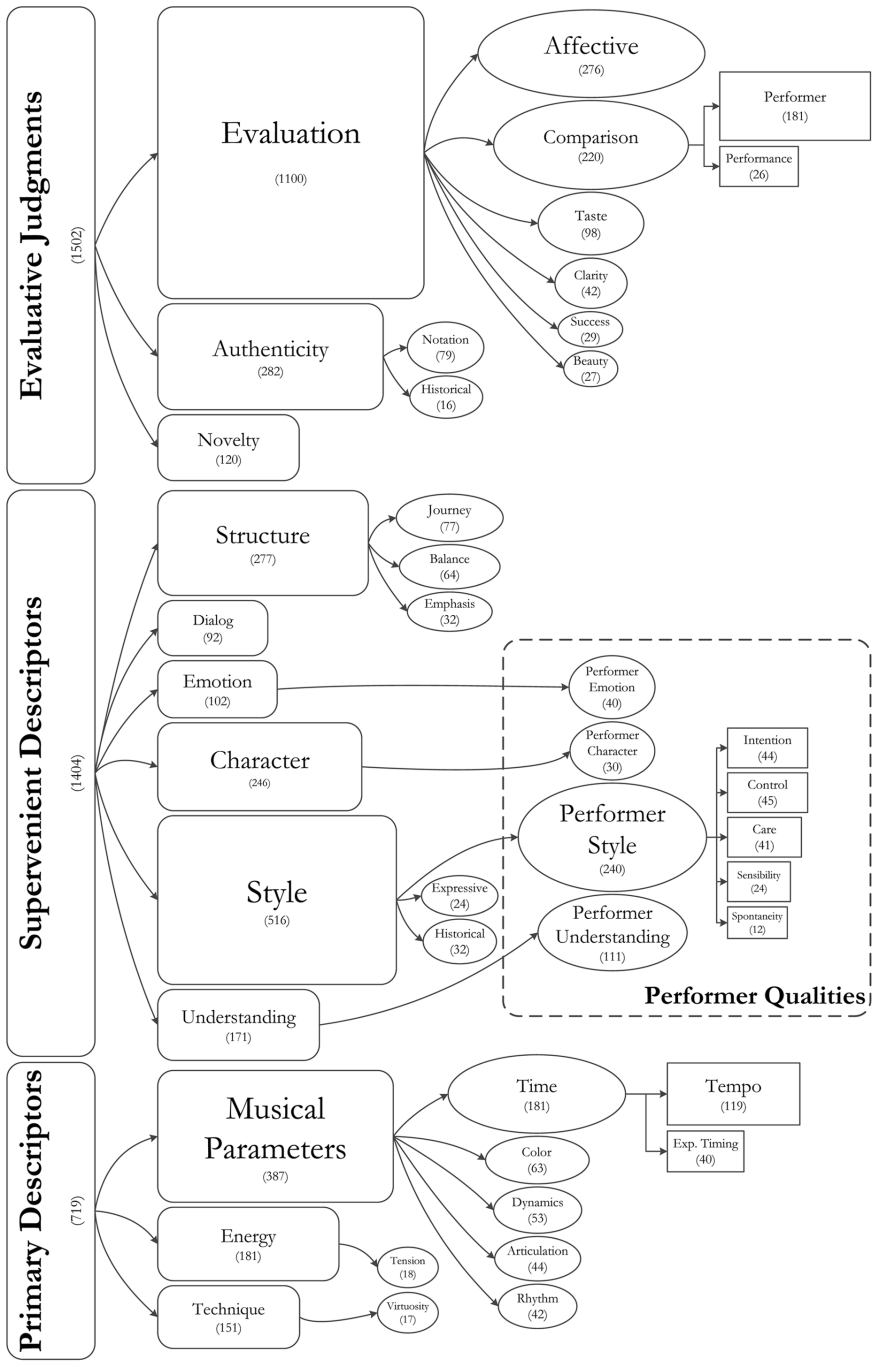
**Performance-related themes discussed by critics.** Superordinate theme families are located on the left hand side of the model. Themes are visualized hierarchically moving from left to right, and from rounded rectangles, leading to oval, and when necessary down to square shapes. Arrows reinforce the visualization of this hierarchical structure. Shape size roughly suggests the relative weight of themes, in terms of frequency of occurrence (not to scale). In parenthesis under each theme name is the number of times the theme was coded in the texts. Each time a sub-theme was coded, the relevant higher-order themes were coded as well.

In the following section, we provide a description for each theme – with superordinate family names in bold, dominant themes in bold italic and sub-themes in italic – together with theme definitions from the codebook and examples from the texts. Issue, page, and critic’s name are given for each example^5^. Numbers in parentheses after theme names indicate the frequency with which the theme was coded in the text. **Figure 1** visualizes the emergent descriptive model, with superordinate theme families located on the left hand side of the figure.

### SUPERORDINATE THEME FAMILY 1: PRIMARY DESCRIPTORS

The first superordinate family (719, lower section in **Figure 1**) encompasses three dominant themes that characterize the performance focusing on specific actions or qualities of the musical sound: ***Musical Parameters**,* ***Technique***, and ***Energy***.

**Musical Parameters (387)**: This large dominant theme entails comments on the nature of the musical sound that can be either local, concerning single notes or phrases, or global, concerning the whole piece, a movement, or a section of movement. Within this dominant theme there are five sub-themes.

*Dynamics* (53) and *Time* (181) comprise comments on loudness and speed (beat frequency and its fluctuations) of the musical sound respectively:

“Brendel has the rare ability to play very quietly and to make the sound rise from almost nothing” (Fiske, August 1963, p. 31)

Comments on *Time* were further divided into *Tempo* (119, global level) and *Expressive Timing* (40, local level, including comments on pause duration):

“The slow movement is surely fastish for a soulful Largo” (Chissell, March 1969, p. 66)

“In the earthy G major Sonata Goode hams up Beethoven’s wit by extending the rests, sometimes by virtually a whole beat” (Fanning, September 1990, p. 116)

*Color* (63) focuses on qualities of sound that relate to timbre and texture:

“… the sonority is more astringent” (Distler, September 2006, p. 80)

*Articulation* (44) focuses on the way in which two successive notes are connected. It includes comments on accentuations as well as technical terms used to indicate ways of connecting notes (staccato, legato):

“… some of his sforzandi, notably in the scherzo, are understated to the point of inaudibility” (Fiske, October 1958, p. 65)

“… the staccato bass is as clearly set off from the legato melody as I have ever heard it” (Chissell, October 1980, p. 71)

The last sub-theme is *Rhythm* (42). Rhythm can be defined as a pattern of accents (see Cooper and Meyer, 1960, p. 6), whose perception is co-determined by other parameters like pitch-contour, articulation, dynamics, or tempo. As such, no assumptions were made about rhythmic content. Passages in this code must have explicitly included the term ‘rhythm’ and its variants:

“The first movement of the A flat has little rhythmic impulse” (Porter, September 1957, p. 17)

***Energy (181)***: This dominant theme captures aspects of the performance that convey energy:

“But in the final resort it is the voltage that counts in this eruptive fugue” (Chissell, March 1972, p. 74)

The only sub-theme focuses on *Tension* (18) and includes comments that entail the term “tension” and related words (tense, release, etc.):

“This is not a reading which is consciously daemonic, fluid or exquisitely ‘painted,’ the tensions eerily depressed” (Osborne, August 1986, p. 49)

***Technique (151)***: In this third dominant theme the critic focuses on the mechanistic qualities of musical delivery. This includes comments on pedaling, hand de-synchronization and repeats and ornaments realization:

“Paik shows off by fingering the prestissimo octaves rather than playing them as glissandi” (Distler, October 2005, p. 81)

“I questioned his ornaments in the second subject. Most people play them on the beat, not ahead of it” (Chissell, June 1971, p. 54)

“… it sounds as if the aura comes from a left-hand chord silently depressed and held down through a series of ‘half-pedals”’ (Porter, November 1956, p. 55)

The only sub-theme within ***Technique*** is *Virtuosity* (17), which collects passages in which the term ’virtuosity’ and its correlates (e.g., virtuoso, virtuosic) are overtly mentioned:

“… he neither subjects the notes to his virtuosic will, nor demeans his own technique by mimetic attempts at audible disorder” (Osborne, December 1983, p. 84)

### SUPERORDINATE THEME FAMILY 2: SUPERVENIENT DESCRIPTORS

Within this superordinate family (1,404) are dominant and sub-themes that portray the performance focusing on higher-order properties than those designated by Primary Descriptors. The largest dominant theme in this group is artistic ***Style***, accompanied by ***Structure***, ***Character***, ***Understanding, Emotion,*** and ***Dialog.*** It is the most varied group of themes and the richest in metaphors and similes (middle panel in **Figure 1**).

Within this large family, a group of sub-themes emerged that focuses on the qualities of the performer, rather than the performance. These four sub-themes (*Performer Style*, *Performer Emotion*, *Performer Character,* and *Performer Understanding*) are presented separately under the heading Performer Qualities.

***Style (516)***: The second largest dominant theme emerging from the full analysis encompasses characterizations of the performance that describe the manner of execution. It includes a large number of terms and expressions used metaphorically:

“The fourth variation is turgid” (Fanning, March 1990, p. 69)

“Casadesus strangely suggests at times a little French acrobat hopping through his paces” (Porter, May 1956, p. 49)

The sub-theme *Historical* (32) focuses on manner of execution linked to different performance practices or historical periods:

“This is the romantic approach to such music” (Fiske, November 1959, p. 68)

“… a sort of Beethoven playing which has surely been outdated since Schnabel” (Porter, June 1954, p. 42)

A further sub-theme characterizes the performance in terms of its *Expressive* (24) content. These passages suggest artistic styles that make use of expressive inflections or that are generally described as expressive (for a detailed analysis of the use of the term ‘expression’ in *Gramophone* reviews see Alessandri, 2014):

“He plays the first page with almost no expression at all” (Fiske, November 1959, p. 68)

***Structure (277)***: This dominant theme includes comments on the way in which the performer portrays the design of the music, its elements, patterns and relationships between them (as well as patterns and relationships that ought to be there but are not realized). It includes comments on phrasing and a substantial number of visual metaphors:

“The tempo is spacious, apt to Gilels’s mastery of the music’s asymmetric lines and huge paragraphs, paragraphs as big as an East Anglian sky” (Osborne, December 1983, p. 84)

“Imagine … Wilhelm Kempff’s clipped phrasing and intimate dimensions, and you’ll get a general sense of Ciccolini’s detail rather than bigpicture-oriented aesthetic” (Distler, April 2007, p. 82)

A sub-theme of structure is *Journey* (77). This includes comments that convey the idea of movement. The portrayal of music is described as a dynamic process:

“His supple unwinding of the Trio is most attractive” (Porter, May 1958, p. 16)

“The Scherzo begins its enchanted journey” (Osborne, February 1996, p. 75)

Another sub-theme of ***Structure*** is *Balance* (64), which focuses on the portrayal of musical design in a coherent, unified or balanced way:

“Wührer has balanced to perfection the component sections of this moderato cantabile” (Porter, June 1957, p. 19)

A last sub-theme is *Emphasis* (32). Here are comments on a portrayal of the musical design that brings to the fore specific elements or details of the music:

“… a more than usually striking significance is given to the four-note phrase so reminiscent of the opening of the Fifth Symphony” (Robertson, October 1935, p. 18)

***Character (246)***: This dominant theme entails characterizations of the performance in terms of mental and moral qualities of an individual or of an atmosphere:

“Balm or solace indeed after the dark and ceremonial Funeral March” (Morrison, May 2005, p. 104)

“His opening to Op. 101 … is suitably devotional” (Morrison, May 2005, p. 104)

**Understanding (181):** Comments on qualities of the performance and its realization that reflect reasoning and use of intellect:

“The brief reminiscence of the opening bars might have been more ruminative” (Fiske, August 1963, p. 31)

***Emotion (102)***: Characterizations of the performance in terms of affective states. The decision about what terms delineate an affective state was made based on the list of stems provided by the Geneva Affect Label Coder (GALC), described in Scherer (2005). In the following examples the word stems rag^∗^, rapt^∗^, and happ^∗^ were used for the coding:

“… what a sense of elemental rage in the heaven-storming finale of the Appassionata” (Morrison, January 2005, p. 76)

“… the latter is more rapt at the very end” (Fiske, February 1961, p. 48)

“… a performance in which the Scherzo successfully reasserts its claim to being the happiest piece of music ever written” (Osborne, November 2000, p. 86)

***Dialog (92)***: Comments on the communicativeness of the performance, as well as speech metaphors:

“There’s a failure of communication somewhere here and I’m just not getting the message” (Plaistow, July 1966, p. 47)

“… a far greater use of declamatory effects and rhetorical tropes than was the case in either of the two earlier cycles” (Osborne, February 1996, p. 75)

#### Performer qualities

Under this heading are grouped sub-themes of Supervenient Descriptors that focus on the player, rather than on the performance. They describe his/her traits or dispositions toward the music and include comments on the *Performer Style*, *Performer Understanding, Performer Character*, and *Performer Emotion*. These comments were found in 93 out of the 100 reviews.

*Performer Style (240)*: ***Style*** describes manners of execution. *Performer Style* entails comments on manner of execution that reflect the pianist’s attitude toward or approach to the work:

“Solomon played this movement with immense reverence as though he thought it the greatest piano music in existence” (Fiske, November 1959, p. 68)

Within *Performer Style* five further sub-themes emerged that focus on *control, intentionality*, *care*, *sensibility*, and *spontaneity*.

*Control* (45) comments on the performer’s aesthetic and technical command of the performance. It also includes comments on the performer’s effort or difficulty in performing:

“I’m bound to acknowledge Gilels’s peerless control over tone, tempo, and phrasing” (Osborne, May 1983, p. 49)

*Intentionality* (44) entails comments on assumed performer’s intentions, preferences and decision processes:

“… here she screws up the tensions of the music (evidently intentionally)” (Plaistow, June 1963, p. 36)

*Care* (41) focuses on the performer’s carefulness in dealing with aspects of the music or performance:

“… one still senses the meticulous, almost pointillist care over each individual note” (Fiske, November 1957, p. 17)

*Sensibility* (24) entails comments on the performer’s ability to appreciate and respond to complex aesthetic stimuli, his/her sensitivity to the presence or importance of certain musical features:

“In Op. 110 he is most exquisitely sensitive to the phrases” (Porter, October 1954, p. 51)

Finally, *Spontaneity* (12) comments on the performer’s deliberation in realizing the music:

“… these five sonatas show that Schnabel’s performances, however deeply considered, emerged fresh and spontaneous” (Morrison, January 2005, p. 76)

*Performer Understanding (111)*: This sub-theme of ***Understanding*** entails comments that reflect the interpreter’s comprehension of the music and his/her discernment or imaginative power in its realization:

“Maria Donska makes her own contribution by playing all three sonatas perceptively” (MacDonald, November 1964, p. 52)

The critic may suggest the performer’s vision of the music, question his/her understanding, or discuss his/her agreement with it:

“I should be most interested to hear an explanation of this interpretative eccentricity” (Robertson, April 1936, p. 18)

*Performer Emotion (40):* This sub-theme of ***Emotion*** focuses on affective states that are construed as qualities of the performer:

“Sheppard revels in the whimsical Menuetto” (Morrison, June 2006, p. 71)

*Performer Character (30)*: This sub-theme of ***Character*** focuses on mental and moral qualities of the performer:

“Bernard Roberts is a Beethoven interpreter of sterling integrity” (Osborne, November 1995, p. 146)

### SUPERORDINATE THEME FAMILY 3: EVALUATIVE JUDGMENTS

The first two theme families comprise judgments that portray aspects of the performance. The last and largest theme family (1,502) focuses on judgments on the value, importance, usefulness or merit of the performance and the performer.

Evaluative Judgments emerged as a pervasive and substantial constituent of critical review with an average of 13.29 occurrences per review (10.36 occurrences/review of Supervenient Descriptors; 6.36 of Primary Descriptors) and a high frequency of co-occurrence with the other families (67.92% of Primary Descriptors and 61.77% of Supervenient Descriptors co-occurred with Evaluative Judgments). This third superordinate family is visualized at the top of the model in **Figure 1** and entails three dominant themes: ***Evaluation***, ***Authenticity,*** and ***Novelty***.

***Evaluation (1,100)***: The largest single theme to emerge from the analysis and the only one present in each of the 100 reviews. It includes judgments about the value or merit of the performance as a whole, of performance temporal segments (e.g., fourth variation) or of performance features, as well as comments on degrees or amounts that clearly delineate a valence of the judgment. These judgments entail little or no descriptive content (superb, bad, to be reckoned with, screws up, too much, unduly). ***Evaluation*** can be expressed in isolation, as a pure evaluative judgment of the performance or temporal fragments of it:

“In total, a fine performance” (MacDonald, May 1981, p. 92)

“Serkin introduction to the final fugue is superb” (Chissell, March 1972, p. 74)

Most of the time, however, they are presented within a sentence as judgments of certain Primary or Supervenient Descriptors:

“Papadopoulos has already scuppered himself with a disastrous drop in tempo for the Fourth Variation” (Fanning, November 1992, p. 152)

“The Adagio, as so often with this player, lacks tenderness and is too heavy” (Robertson, April 1936, p. 18)

The largest sub-theme within ***Evaluation*** includes *Affective* (276) judgments that reflect perceptions of the performance or its features, focusing on the listeners’ affective reaction (breath-taking, horrifying):

“I have never heard this done before and it is strangely moving” (Robertson, August 1950, p. 23)

“… some may find Schiff’s arpeggiation of the second theme cloying” (Distler, December 2005, p. 97)

Also, comments reflecting perceptions of the performance which aim to add to the listener’s understanding of the music:

“Angela Hewitt … offers intelligent, stylish and often illuminating interpretations” (Distler, November 2006, p. 97)

Another large sub-theme is *Comparison* (220), in which judgments are made in relation to another performance by the same or a different pianist:

“Though not quite up to Arrau’s, he plays the so-called Les Adieux sonata most beautifully” (Fiske, November 1959, p. 68)

A further sub-theme of ***Evaluation*** is *Taste* (98). Here judgments are presented as the critic’s personal perception. These comments, present in 47 out of 100 reviews (see **Table 2**), tend to be holistic, thus focusing on qualities of the performance at global level:

“I invariably find myself won over by Ashkenazy in this sonata – no matter what formidable counter claims have come before. Yet chacun à son goût” (Chissell, March 1972, p. 74)

Finally, three minor sub-themes within ***Evaluation*** focus on judgments of *Clarity*, *Success, and Beauty*.

*Clarity* (42) relates to either technical qualities of the performance or structural clarity with which the music is portrayed:

“… the clarity of the toccata-like part writing and off-beat accents make Brautigam’s conception work” (Distler, December 2008, p. 103)

“But it is Brendel who gives you the clearer semiquavers in bar 3” (Chissell, December 1970, p. 86)

*Success* (29) focuses on the performance as product of the pianist’s achievement:

“It seems to me that Miss Donska here succeeds without a doubt” (MacDonald, November 1964, p. 52)

*Beauty* (27) was coded only when the critic used the word ‘beauty’ or a variant:

“The fugue is beautifully done – particularly the reprise” (Porter, June 1957, p. 19)

***Authenticity (282)***: This is the second dominant theme within the **Evaluative Judgments** family. It entails comments built on assumptions about the composer’s thoughts, the period style, and in general the existence (or not) of a valid or correct interpretation of the given work:

“Only in the Coda does Beethoven himself seem to speak for a moment” (Robertson, April 1936, p. 18)

“He plays the jolly little scherzo and the difficult finale with much virtuosity – the right and only way” (Robertson, April 1936, p. 18)

Discussion often focuses on the correspondence between the performance and inherent qualities of the music that ought to be realized and that the performer did or did not bring out:

“… the spirit of the music has been exactly caught” (Chissell, August 1963, p. 31)

A sub-theme within ***Authenticity*** is *Notation* (79), discussing the correspondence between performance and score indications:

“Taub anticipates the meno allegro indication by a couple of bars and I wish he didn’t apply the brakes quite so soon” (Plaistow, March 1988, p. 50).

Another sub-theme, *Historical* (16), discusses the performance in relation to the context of the work’s composition or the assumed composer’s intentions:

“I am not sure the effect can ever succeed on a modern instrument, but at least Richter-Haaser’s attempt is nearer the composer’s wishes than Kempff’s and Backhaus’s total rejection of the sustaining pedal” (Fiske, February 1961, p. 48)

***Novelty (120)***: This final dominant theme encompasses characterizations of the performance or of its features that reflect originality. It also includes comments that refer to the originality of the pianist as an interpreter, highlighting interpretative style:

“Arturo Pizarro now re-emerges on Linn Records with performances of four Beethoven sonatas sufficiently individual and freshly conceived to make them emerge as new-minted rather than over-familiar” (Morrison, March 2003, p. 63)

### CONSISTENCY IN THE RELATIVE USE OF THEMES

The previous sections have described the map of performance-related themes that emerged from the analysis of the 100 selected reviews. These themes indicate aspects of performance that critics discuss in their judgments. A major concern within research investigating the perception and appreciation of music is the extent to which judgments may be shared between people, or the extent to which different listeners may normally focus on those same aspects of performance (Kinney, 2009). An important aspect of the emergent model is thus the level to which it can be taken as representative of a common trend among different critics.

Differences between critics in the relative use of themes may be linked to personality, musical background, and reviewing and linguistic style. However, one additional important factor should be taken into consideration in the present analysis. The corpus of critical review at hand entails reviews of Beethoven’s piano sonata recordings. Each review discusses a different disk or set of disks. Looking for differences and commonalities between critics in the use of the emergent themes thus means comparing reviews that discuss different performances, most often of different musical works. A performance that lacks, say, rhythmic stability might then trigger comments on the rhythm that may not be necessary in a performance being technically impeccable but lacking in energy.

These influences (reviewed object, personality, writing style, and musical background) are compounded in the material and cannot be taken apart in the present study. What can be examined though is the extent to which, notwithstanding these confounding factors, the relative weight given to each theme is consistent between reviewers. Keeping this in mind, and in line with the results of the data reduction analysis run on the review vocabulary (iv), it is reasonable to expect a certain amount of variety across the critics.

**Table 2** shows the distribution of dominant and first level sub-themes across the 100 analyzed reviews and for each critic separately, with themes treated as dichotomous variables: the frequency shows for each critic the number of reviews in which the theme occurred at least once. All twelve dominant themes and a third (36.36%) of the first level sub-themes were reflected in the writings of each of the ten analyzed critics.

**FIGURE 2:**
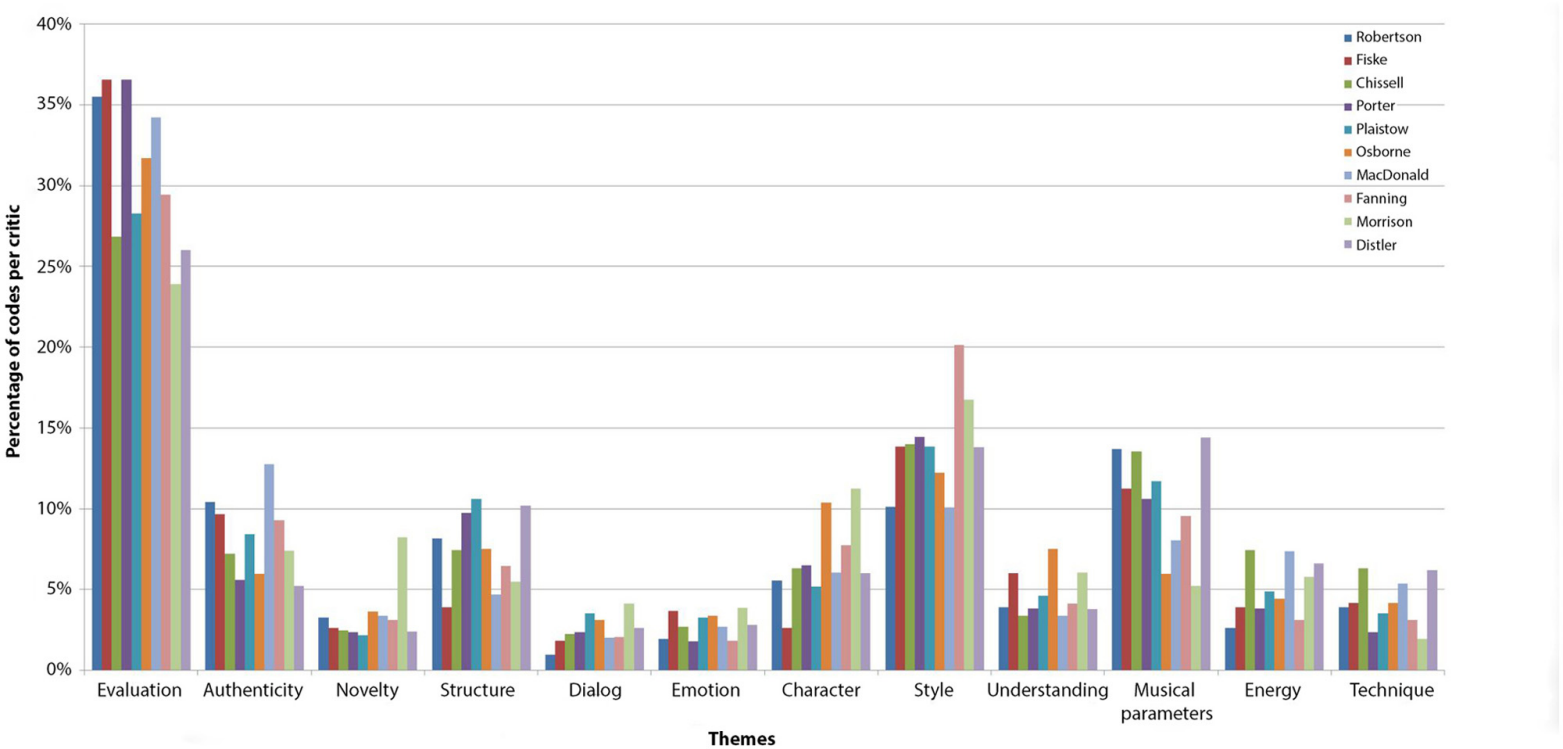
**Distribution of codes across dominant themes for each critic.** For each critic, the relative frequency is shown with which each theme was coded in the text.

**Figure 2** shows then the relative frequency with which each dominant theme occurred in the reviews of each critic. To partially compensate for the variety due to different performances and musical works reviewed, code occurrences for each critic across the ten reviews were added together. Following Simonton (2004)^6^, Cronbach’s alpha was computed as a measure of internal consistency in the relative use of the 12 dominant themes between critics. This showed a high level of agreement, α = 0.986.

The data reduction analysis run on the review vocabulary [analysis (iv)] showed, however, a high level of variability in the terms used by one and the other critics. This variability does not seem in line with the internal consistency found in the use of themes. One possible explanation is that critics’ writings, despite showing distinctive word patterns, shared the core underlying musically meaningful constructs. The level of text understanding necessary to capture these constructs was beyond what could be achieved through a single-word text content analysis.

Furthermore, care should be taken in comparing the two results, given the different analyses used. Variability in the vocabulary between critics reflects differences in the use of single semantic categories. The consistency found in the relative use of themes on the other hand indicates that the emergent cluster of themes, the model as a whole, captures a general pattern among the observed critics.

## DISCUSSION

The purpose of the present study was to understand the content of one of the most common yet least understood written responses to music performance: critical review. We employed a novel data reduction technique and inductive method for in-depth qualitative text analysis. The extensive analysis and subsequent emergent visual model categorizes the content of 100 reviews (35,753 words) of Beethoven piano sonata recordings written by 10 of the most prolific reviewers of classical repertoire from the *Gramophone* magazine. The results reveal, for the first time, the content of a large corpus of music criticism. In so doing, the findings both confirm but also challenge accepted wisdoms regarding the nature of art criticism.

The research question for the present study was: what do experts write about when reviewing a recorded musical performance? The answer can be summarized in terms of properties of the musical sound, level of energy, and mechanics of musical delivery (Primary Descriptors), higher-order impressions of the performance (Supervenient Descriptors), and the value that any of these properties, or combinations thereof, possess (Evaluative Judgments). As such, the emergent visual model supports the view of music critical review as a form of reasoned evaluation (Beardsley, 1968).

An important point is that the present model – resulting as it does from the analysis of the end-product of the critical process – does not allow us to determine if critics’ evaluations are inferred from Primary and Supervenient Descriptors (performance X possesses properties A, B, and C, therefore it is good) or simply connected to them (evaluation comes as an instinctive response, and then reasons are sought to explain this response). Further studies, focusing on the temporal component of the critical process, are needed to address this issue.

The different performance properties (Primary and Supervenient) identified in the model, though differently organized, reflect musical factors commonly used in performance assessment in music education (McPherson and Schubert, 2004, pp. 63–64) and therefore concur with previous literature in this area (Fiske, 1977; Wapnick et al., 1993; Bergee, 1997, 2003; Thompson and Williamon, 2003; Kinney, 2009).

One criterion commonly used in assessment schemes is musical expression. There is evidence for the presence of expression in the present model, though not as a distinct theme. ‘Expression’ is among the most discussed topics in musical parlance yet there remains no unanimous understanding of its meaning (Lindström et al., 2003). A previous investigation focused on the notion of expression in music criticism (Alessandri, 2014) found that critics use this term, and its correlates, to indicate at least four different properties of performance: specific actions or qualities of the musical sound; ways of portraying the musical design; communication of higher-order qualities (such as emotions); or as an undefined, positively loaded evaluation. All these elements are represented in the present study’s visual model, but not as one cohesive theme. This is likely due to the fact that the data analysis was emergent from and grounded in the text data. Critics use the term ‘expression’ and its correlates rarely (***Style***_*Expressive*) therefore no *a priori* assumption was made on the nature of this multi-layered and ambiguous notion. As such, the properties of expression emerged from the data but interconnected with other larger themes (***Musical Parameters***,*** Structure***,*** Emotions***,*** Character***,*** Style***, ***Evaluation***).

One trait of critical review that is atypical of music written responses in education settings is evaluative judgment that depends on a listener’s personal perception and preferences. This trait is represented in the visual model by the sub-theme *Taste*. The co-existence in reviews of absolute and relative (taste-dependent) evaluative judgments resonates with findings from performance assessment research (see Thompson, 2009, p. 205) and Levinson’s (2010) statement that judgments of value cease to be meaningful at a certain level, and beyond this point individual preferences become a decisive choice criterion. In a similar vein, critics’ judgments also focus on ***Novelty***, artistic ***Style,*** and the *Affective* response of the listener.

Overall, the present analysis has revealed a degree of overlap in the content of critical review and aspects that drive written response to music performance in education settings, while still identifying properties that are distinctive of professional critical review. Even if not directly transferable, considerations on the content of critical review may be used to stimulate discourse on assessment related topics such as the importance of comparison and questions of taste among music students and pedagogues. Performance evaluation in general is a complex and often unclear terrain. Expert critics have developed a currency of terms they use to navigate on this ground. Teaching students and musicians how critics write and what they look for in performances will help to pass this knowledge on to them, giving them new vocabularies and conceptual tools to be used in their preparation for performance and reflections upon their own practice.

The present study permits wider reflection on the use of different aesthetic criteria in critical review. Van Venrooij and Schmutz (2010) listed indicators of high art vs. popular aesthetic criteria derived from the literature as part of their study of popular and classical reviews. Indicators of high art criteria included discussion of context, the performer as creative source, comparisons with high art (masterworks), originality, complexity, seriousness, and timelessness. By contrast, indicators of popular aesthetics included participatory experience (rousing, catchy) and the use of language related to “primary” tastes, like oral and food-related metaphors (pp. 405–406). This dual categorization can also be found in the present corpus. Comparisons between interpretations and performers (high art) were found in 63 of 100 reviews, in line with Alessandri et al. (2014). However, popular aesthetics judgments were also common, such as those that indicate listener responses to the music (*Affective*: 89 of 100 reviews). Thus, following Van Venrooij and Schmutz (2010), it can be concluded that the present corpus of classical music critical review provides a combination of high art and popular aesthetics for the reader.

Another wider issue of concern to the present study was the debate regarding the importance of evaluation in critical review. In fact, ***Evaluation*** was the largest theme in terms of absolute frequency of coding and spread among reviews, found to permeate critics’ judgments of performances in the present model. ***Novelty*** and ***Authenticity*** were also widely spread and presented further evaluative dimensions. These results reflect the importance of evaluation in music critical review (Calvocoressi, 1923; Newman, 1925; Walker, 1968; Cone, 1981; Carroll, 2009). This finding does not concur, however, with the results obtained by Conrad et al. (2005), who found that less than half of 181 music critics saw evaluation as an important element in their writing. An explanation for this apparent discrepancy lies in the variety of music critical activities. Among the critics surveyed by Conrad et al. (2005) 53% stated that half or more of their writings were “profiles of musicians, composers and musical figures” (p. 16). In line with this, 41% of critics defined themselves not as critic, rather as “arts reporter,” “music writer,” “program annotator,” “general assignment critic,” or “entertainment writer” (p. 12).

Seen in this light, the general debate on the nature of evaluation in art criticism is limited by factors such as different media (general newspapers vs. specialist magazines) and art domains (music vs. visual arts; and within music, live vs. recorded performances). For instance, Danto’s view of art criticism as a descriptive rather than an evaluative practice (Rubinstein, 2006) may reflect the fact that visual art and music consumers are not subject to the same immediate burden of possible purchase choices, thus visual art consumers may not require critics to act as guides for this purpose.

In sum on this point, the results of this study suggest that evaluation is a major component of classical music critical reviews of recorded performances. This article offers a detailed method by which other forms of written response may be analyzed and compared in a similar way, including alternative musical genres or art forms. Such future investigations will permit a better overview of the variety and importance of an evaluative component in different forms of art criticism.

Finally, an unexpected result from the present analysis of critical review was the focus on presumed qualities of the performer. This finding – reflected in the themes grouped under Performer Qualities – suggests that the musical agent perceived behind performance actions plays an important role in the appreciation and interpretation of a performance. This is despite the fact that these comments are based on assumptions about the performer which, in the case of the present recordings, the critic could not even see.

This result is in line with theories of the role of intentionality from the philosophy of art. Levinson (1996) argues that a person cannot reliably interpret performance actions as reflecting the critical conception of the artist, since no one-to-one correspondence can be established between the two. Nonetheless, such thoughts are common, playing an important role in our understanding and appreciation of the music. In his discussion on the interpretation of artistic works Currie (1993) calls this process ‘intentional explanation’ (p. 416), ascribing intentions to the artist such that his/her behavior is viewed as depicting his/her intentions. According to Currie (1993), intentional explanation allows us to create a coherent narrative of the work and is thus essential to our understanding. Carroll (2009) also claims that when we evaluate a performance one of the things we judge is the performer’s achievement – ‘success value’ (p. 53). To assess this aspect we need to know what the artist intended to achieve, how ambitious his/her intentions were, what risks s/he had to take, and so on.

The important role of the perceived musical agent has implications for the understanding of the listening experience in general. In recent years there have been notable efforts in the development of computer systems for expressive music performance (for a review see Bresin and Friberg, 2013; Kirke and Miranda, 2013). However, the results of the present study of critical review confirm that the opportunity to entertain thoughts concerning the *persona* behind the performance, his/her will, decisions, emotional state, and moral qualities, remains a significant part of the music listening experience.

In conclusion, for this study we assembled a representative sample of recorded performance critical review in order to examine its content. A novel combination of data reduction and thematic analysis techniques were employed to derive and categorize the text corpus. Despite the focused nature of the data source (*Gramophone* magazine), musical format (recorded performances), and repertoire (Beethoven’s piano sonatas), the corpus was dense in information content and has offered new insights relevant to our understanding of expert performance evaluation and art criticism in general.

These insights provide solid empirical grounds for the development of future testable hypotheses. Music critical review represents a vast heritage of rich source material that has been barely touched upon until now. The present method and model provide the basis for examining other forms, corpuses, and aspects of critical review. In particular, comparison of the present findings with investigations of reviews published in different musical and cultural contexts and concerning a different repertoire would be necessary to highlight common traits that may form a more general model of critical review and to offer insights on the components that are context-specific.

At present, the highly dense nature of critical review writing in the chosen sample did not allow the analysis to be moved beyond the level of theme description to explore patterns between different themes. A further step to this research should then attempt to explore how Evaluative Judgments are connected to the different kinds of properties referred to by the Descriptors in reviews, thus offering insights on the valence of critics’ judgments and the criteria underpinning their evaluations. Such studies would add to the insightfulness and generalizability of the present model and further our understanding of this well-established, authoritative and highly relevant form of written music response.

## Conflict of Interest Statement

The authors declare that the research was conducted in the absence of any commercial or financial relationships that could be construed as a potential conflict of interest.

## Footnotes

1 The version used in this analysis was 0.99835, available from http://GKing.Harvard.Edu/readme under the Creative Commons Attribution-Noncommercial-No DerivativeWorks 3.0 License.

2 Analysis (i) was run for each decade separately. Results are reported in Alessandri et al. (2011) and show an increased focus on interpretative issues over the course of the century. Concerning analysis (ii), a validation task run on the ReadMe algorithm (employing data from analysis i) showed an RMSe at 3.56% points. This suggested that a single decade analysis would be too finely grained, requiring a level of accuracy of the software estimate that could not be assumed here. Thus, analysis (ii) was run for the periods 1923–2010, 1923–1950, and 1991–2010 only, to confirm results of analysis (i).

3 In the latter group eleven categories emerged: achievement, musical parameters, structural details, expression, originality and insights, intensity, ideal interpretation, unity, accuracy, variety, clarity.

4 For each review, all sonatas entailed in the reviewed recording were counted, notwithstanding the critic discussing or not explicitly the single pieces.

5 Original review texts can be accessed through the *Gramophone* online archive, currently available as paid application for iPad, desktop or tablet. See http://www.gramophone.co.uk.

6 Simonton applied Cronbach’s alpha to test internal consistency in the adjudication of awards and nominations between seven film award organizations (including the Academy of Motion Picture Arts and Sciences). The coefficient was taken as a measure of the agreement between the seven organizations on the cinematic contribution of a given film. In the present analysis, the alpha coefficient is used as a measure of agreement between critics on the weight (in terms of frequency) given to a certain theme.
